# Molecular Drivers of Potential Immunotherapy Failure in Adrenocortical Carcinoma

**DOI:** 10.1155/2019/6072863

**Published:** 2019-04-01

**Authors:** Chiara Fiorentini, Salvatore Grisanti, Deborah Cosentini, Andrea Abate, Elisa Rossini, Alfredo Berruti, Sandra Sigala

**Affiliations:** ^1^Section of Pharmacology, Department of Molecular and Translational Medicine, University of Brescia, Viale Europa 11, Brescia, Italy; ^2^Oncology Unit, Department of Medical and Surgical Specialties, Radiological Sciences, and Public Health, University of Brescia and ASST Spedali Civili di Brescia, Piazzale Spedali Civili 1, Brescia, Italy

## Abstract

Adrenocortical carcinoma (ACC) is a rare, highly aggressive cancer, often insensitive to conventional chemotherapeutics agents. Early diagnosis, followed by radical surgical resection plus/minus adjuvant mitotane therapy, is nowadays the only valuable option. Unfortunately, one out of four patients has metastatic disease at diagnosis and most of radically resected ACC patients are destined to recur with local or metastatic disease. Numerous efforts aimed at identifying molecular alterations crucial for ACC pathogenesis have been extensively conducted, with the hope to develop new treatments. Indeed, multiple genes and pathways have been identified as potentially targetable in ACC patients; however, despite the strong preclinical rationale, translational findings to clinical trials led to date to disappointing results. The immunotherapeutic intervention targeting T-cell checkpoint molecules has been proposed as well, but results obtained in early studies indicate that ACC patients would be unlikely to benefit from immunotherapy. Genetic alterations of different pathways involved in ACC carcinogenesis are also known substrates of resistance to immunotherapy. Among them, *β*-catenin gene CTNNB1 and TP53 gene are frequently mutated in ACC samples. Overactivation of the *β*-catenin pathway and loss of p53 protein function are potential tumor-intrinsic factors that, impacting on the ability of ACC cells to recruit dendritic cells, leading to T-cell exclusion, put this tumor among those that are potentially resistant to immunotherapy. Moreover, the steroid phenotype, which implies glucocorticoids hypersecretion in a subset of ACC, contributes to generating an immunosuppressive microenvironment. Here, we review clinical results of immunotherapy in ACC and we highlight molecular mechanisms driving immunotherapy failure in ACC, suggesting possible approaches to overcome resistance.

## 1. Background

Adrenocortical carcinoma (ACC) is a rare tumor derived from the adrenal cortex, with an estimated incidence between 0.7 and 2.0 per million population per year. ACC could occur at any age, but the peak of incidence is between 40 and 60 years, with higher prevalence in female (up to 60%) [1]. Despite intense efforts to improve management of ACC, both with preclinical and clinical studies, prognosis remains overall limited, although it has been recently recognized that ACC is a very heterogeneous disease and harbors a variety of morphological, clinical, and genetic variants that have a prognostic value [2, 3]. ACC is mostly sporadic, although it can be diagnosed within hereditary syndromes, such as Li-Fraumeni and Lynch syndromes, associated with specific germline mutations in TP53 gene or in various mismatch repair genes, respectively [4].

The disease stage at diagnosis is a key prognostic factor for ACC: 5-year survival for 60-80% in patients with stage I, up to 50% for locally advanced disease, dropping to a very low percentage (0-28%) in the case of metastatic disease [1]. Other prognostic factors at diagnosis are proliferation activity [5] and cortisol hypersecretion [6]. In regard to the management of ACC, current guidelines [1, 7] recommend the complete surgical resection of primary tumor as the only potential curative treatment, although it is a realistic approach only in patients at stage I and II of disease and more rarely in those at stage III. In patients radically operated however, recurrence frequently occurs (30%-70% of cases) [1]. This is the reason why adjuvant mitotane, an adrenolytic drug [8], is prescribed in the majority of patients [1], although the efficacy of this drug in the adjuvant setting is supported by the results of a retrospective multicenter international study showing that postoperative mitotane treatment is associated with a significant reduction of the risk of relapse and death [9]. The management of patients with metastatic or inoperable disease (45% of patients at diagnosis [1]) requires systemic treatment which consists in either mitotane alone or mitotane plus etoposide, doxorubicin, and cisplatin (EDP-M) [10, 11]. Mitotane is the only drug approved to treat ACC, but its role is conditioned by the possibility of attaining therapeutic concentrations in the plasma [12]. However, its pharmacokinetics, safety profile, and adverse effects show high interindividual variability and strongly limit its efficacy [13]. The great majority of patients who received mitotane and EDP-M are destined to undergo disease progression. In these patients, a pharmacological approach that includes gemcitabine with capecitabine could be administered [14, 15], but this regimen has a limited clinical benefit. On these bases, there is a need of new treatment strategies.

In the past few years, molecular characterization of ACC identified genetic and molecular abnormalities and disclosed novel potential druggable molecular targets to develop new therapies. In particular, several comprehensive analyses of the genomic profile of ACC have been performed, showing a complex genomic landscape with the identification of recurring mutations in different genes such as ZNFR (20%), CTNNB1 (14%), TP53 (14%), and RB1 (11%) [3, 17, 18]. As previously mentioned, various genetic alterations are present in subgroups of tumors with different clinical characteristics and outcomes; of relevance, mutations in the CTNNB1 gene encoding for *β*-catenin and in the TP53 gene encoding for the tumor suppressor p53 proteins have been defined as poor prognostic factors for ACC [19]. Moreover, both mutations in CTNNB1 and TP53 genes have been shown to play a role in ACC carcinogenesis, as early and late events, respectively [20–22]. The Cancer Genome Atlas – Adrenal Cortex, that analyzed 91 cases for alterations in the ACC genome, reveals that, beside the cited molecular alterations, mutations in the PRKAR1A gene (8%) and the overexpression of IGF2 (90%) [3] can be observed as well. Analysis of results further indicates that copy number alterations likely play a critical role in ACC. Whole genome doubling is indeed observed in about 51% of ACC samples; in addition, hypoploidy or the loss of a significant amount of the genome is found in a high number of cases. The study further showed that the frequency of copy number changes is associated with an aggressive clinical course of the disease, supporting whole genome doubling as a key point of disease progression [3]. At the molecular level, in addition to p53 and Wnt-*β*-catenin pathways for which no specific molecular target agents are currently in use, other potentially druggable pathways have been identified in ACC patients, such as the Epidermal Growth Factor Receptors (EGFRs), Insulin Growth Factor-Receptor 1 (IGF-1R), and Vascular Endothelial Growth Factor Receptors (VEGFRs). However, in spite of the preclinical rationale, clinical trials testing drugs targeting these pathways led to disappointing results [23, 24].

## 2. Current Immunotherapy Trials in ACC

Current most successful immunotherapies against cancer are based on blocking key regulators of T cells (T-cell checkpoint molecules). These are inhibitory molecules with the ability to limit immune responses against tumors cells, such as Cytotoxic T-Lymphocyte Antigen 4 (CTLA4) and programmed death receptor 1 (PD-1) or its ligand PD-L1 [16].

Multiple ongoing clinical trials have been designed to explore the role of anti-PD-1 or anti-PD-L1 drugs in numerous cancers, including ACC [25] (Table 1). However, initial clinical findings seem to be unsatisfactory, indicating that ACC patients would be unlikely to benefit from immunotherapy.

In particular, in the JAVELIN international, multicenter phase Ib trial, safety, pharmacokinetics, and clinical activity of the anti-PD-L1 monoclonal antibody avelumab were tested in patients with different metastatic solid tumors (ClinicalTrials.gov Identifier: NCT01772004). In this study, 50 ACC patients, previously treated with mitotane or platinum-based chemotherapy, were treated with avelumab (10 mg/kg IV, every 2 weeks) until progression, unacceptable toxicity or withdrawal. Results demonstrated, as expected, an acceptable safety profile, especially in patients with limited pretreatment. However, only a modest clinical activity was observed since a partial response was obtained in 3 of 50 patients (6%), while 42% and 46% of patients experienced stable disease and disease progression, respectively [26]. More importantly, however, the median progression-free survival and the overall survival of this patient population were 2.6 months (95% CI 1.4–4.0) and 10.6 months (95% CI 7.4—not estimable), respectively. Taken together, these results are similar to those obtained with the previously mentioned second-line combination regimen, gemcitabine and capecitabine. So, at least with the strategies available to date, immunotherapy seems to be not able to improve the current standard therapy in ACC.

## 3. Immunologic Properties of ACC

Several observations contribute to explaining the poor response of ACC to standard immunotherapies.

### 3.1. Immune Checkpoints Molecules

In order to preselect cancer patients most likely to benefit from immunotherapy, PD-L1 expression has been considered and likely reflects an immunoreactive tumor microenvironment [27]. A correlation between tumor PD-L1 expression and response to PD-1 therapy, in fact, has been provided for various cancer types, including melanoma, non-small-cell lung carcinoma, and renal cell carcinoma [27]. Accordingly, PD-L1 expression was investigated in 28 ACC tissues by immunohistochemistry, showing that a small percentage of tumors (10.7%) are positive for PD-L1 expression with a cut-off level of 5% [28]. Therefore, according to this tumor intrinsic parameter, ACC could be poor, if not responsive to immunotherapy. However, the role of PD-L1 tumor expression as predictor of immunotherapy response is currently debating, due to positive ORRs reported in clinical studies in patients carrying PD-L1 negative tumors (ACC cases not included) [29]. For instance, nivolumab treatment is associated with clinical benefits in a number of tumors regardless of PD-L1 expression [29]. Taken together, these findings indicate that PD-L1 expression may not be the ideal biomarker of sensitivity also in ACC and that other markers of clinical efficacy and safety need to be identified.

### 3.2. Glucocorticoids

Another element of intrinsic immunoresistance in ACC is linked with hypercortisolism-secondary immune defects. Patients with cortisol-secreting ACCs are indeed characterized by suppression of T cell activity [30] and altered levels of circulating lymphocytes [31]. Recent insights into genomic characterization of ACC identify the so-called “steroid phenotype” based on differential expression of steroid synthesis pathway genes and clinically translating into patients with glucocorticoids hypersecretion. This subset of ACCs displays the lowest pathological immune scores in cancer stromal cells infiltrates among different human neoplasms. The clinical phenotype of ACC with steroid phenotype also correlates with the lowest overall survival. However, it must be noted that this lethal phenotype is also associated with a high proliferative score indicating that the immunosuppressive microenvironment induced by steroids contributes in part to determining the poorer prognosis, but that the intrinsic aggressiveness of these tumors also depends on other genomic alterations (see below) linked to proliferation [3]. Establishing the exact role of these two components in producing the patient clinical phenotype is beyond the scope of the present review.

Beside endogenous steroid hypersecretion in functioning ACCs, glucocorticoids are frequently prescribed as supplementation to treat adrenal deficiency in ACC patients treated with mitotane or following adrenal surgery. However, doses of steroid replacement and types of steroid used are much lower and different, respectively, compared to what is used for immunosuppressive or anti-inflammatory steroidal therapies [1]. Importantly, an ACC patient should not be excluded from immunotherapy trials because of steroidal replacement therapy nor glucocorticoids should be stopped in case of adrenal insufficiency.

With regard to immunotherapy, it should be kept in mind that despite the immunosuppressive “milieu” induced by glucocorticoids, dominant underlying biological properties of ACC tumors mostly contribute to the dismal prognosis of patients, wrongly inducing the impression of failure of immunotherapy because of glucocorticoids.

### 3.3. Genomic Alterations

The rationale to explain why ACC displays resistance to immunotherapy could be linked to the above-mentioned molecular alterations highly prevalent in ACC, namely, mutations in TP53 and CTNNB genes [17, 32, 33]. It is well known that despite the lymphocytic activation by checkpoint inhibitors, lack of spontaneous T-cell infiltration (non-T-cell-inflamed tumors) might result in immunotherapy ineffectiveness [16]. Interestingly, while the presence of multiple chemokines, such as the CXCL9 and the CXCL10, directly correlates with high number of infiltrating T cells [34], the specific lineage basic leucine zipper transcriptional factor ATF-like 3 lineage of dendritic cells (BATF3 DC) are considered the major source of these chemokines. Therefore, BATF3 DC appear to play a central role in orchestrating antitumor T-cell responses [16]. Several oncogenic pathways have been found to influence the local antitumor immune response by modulating BATF3 DC recruitment; among them, overactivation of *β*-catenin pathway has been associated with a reduced recruitment of BATF3 DC into tumor, leading to failure in chemokine release [35]. Evidence that upregulation of Wnt/*β*-catenin signaling is associated with T-cell exclusion has been provided for metastatic melanoma [35], bladder, head and neck [36], and colorectal cancers [37]. In addition to the Wnt/ *β*-catenin pathway, the inactivating mutations TP53 have been associated with defects in the ability of tumor cells to produce key chemokines required for BAFT3 DC recruitment [38]; p53 loss of function and lack of T-cell infiltration have been found in basal-like ER-negative breast cancers, but not in ER-positive breast cancer [39].

Therefore, either overactivation of the Wnt/*β*-catenin pathway or loss of p53 is potential tumor-intrinsic factors that, altering on the ability of ACC cells to recruit BAFT3 DC cell and leading to T-cell exclusion, likely indicate that this type of tumor is potentially resistant to immunotherapy. This point is strengthened by results reported on CTNNB1 expression and T-cell infiltration that have been investigated in a series of ACC tumors, showing that the increased CTNNB1 expression correlated with reduced infiltration in T cells [40]. Interestingly, high levels of CTNNB1 expression have been associated as well with increased cortisol levels [40] that likely contribute to the clinical resistance of ACC to immunotherapy [41].

Dysfunction of p53 due to mutations may contribute not only to carcinogenesis, but evidence indicates that it may also contribute immunologically to tumorigenesis and tumor progression, altering as well the immune-mediated response in the microenvironment. Indeed, in cancer cells with p53 dysfunction, restoring wild-type p53 drives immunological activity towards antitumor response [42]. Accordingly, in acute myeloid leukemia, there was also a significant increase in PD-L1 expression in patients with TP53 mutations when compared to wild-type TP53 patients [43]. Although these observations were made in tumors other than ACC and this hypothesis needs to be confirmed, it could be suggested that targeting immune escape mechanisms could establish sensitivity to the checkpoint inhibitors in ACC. Thus, combining the administration of immune checkpoint inhibitors with drugs targeting the Wnt-*β* catenin and TP53 pathways could be an attractive treatment paradigm to be explored.

## 4. Strategies to Overcome Immunotherapy Resistance in ACC

The combination approach suggested above could be intriguing in trying to overcome resistance in ACC. Unfortunately, despite several intensive studies, targeting both Wnt/*β*-catenin and p53 pathways is nowadays challenging, due to their important role in different physiological processes, which implies toxicity in case of effective inhibition. In regard to pathways, both active inhibitors of Wnt secretion and Wnt/receptor interactions, including antibodies and small peptides, are being tested in early-phase trials [44] and others are in preclinical development (for a review, see [45]). Among them, the OMP-54F28 agent, a fusion protein comprised of the cysteine-rich domain of frizzled family receptor 8 fused to the human immunoglobulin Fc domain, is able to bind to all Wnt ligands blocking Wnt signaling [46]. Moreover, LGK974, an inhibitor of the porcupine membrane-bound* O*-acetyltransferase, required for posttranslational acylation of Wnt and its subsequent secretion [47], inhibits Wnt signaling both in vitro and in vivo in different animal models [48]. A phase I trial to evaluate safety of LGK974 is ongoing [44]. Another phase I clinical trial, investigating toxicity and activity of the small-molecule CWP232291 that targets *β*-catenin degradation, is currently ongoing in the management of acute myeloid leukemia patients [44].

The above-mentioned drugs, however, are in their early phases of clinical development; thus, they will not be available soon. Thus, an approach to overcome the ACC resistance to checkpoint inhibitors could come from drugs already marketed for other therapeutic indications and that are endowed, as ancillary mechanism, with the ability to target this pathway. Preclinical experimental models could be strategic to shed light on this field. In line with this, we recently demonstrated that in the widely used ACC cell model, namely, the NCI-H295R cells, characterized by an abnormal *β*-catenin nuclear accumulation [49], both the CYP17A1 inhibitor abiraterone acetate that induces an increase of progesterone levels and progesterone itself induce cytotoxicity and partially reduce the nuclear accumulation of *β*-catenin [50, 51]. We are aware that this result, now under a deeper molecular characterization, was obtained with a preclinical in vitro approach, and we would like to stress on the fact that the clinical translation is not obvious. This observation, however, could stimulate further research in this direction, demonstrating as well the possible contribution of the Wnt/*β* catenin in the resistance to immunotherapy of ACC.

Concerning TP53, as already mentioned, it represents the most commonly mutated gene in cancer [52], leading to a great variability on the effects of mutation on p53 activity. Therefore, targeting functional variant mutant p53 requires a mutation-specific approach, ranging from the restoring of wild-type activity of the mutant p53 to the degradation of mutant protein [52, 53]. In ACC, TP53 mutations lead to the production of p53 protein that lacks its physiological function, appearing mostly in the late phase of tumor progression and associated with a poor outcome [2, 54]. Efforts in designing short synthetic peptides able to stabilize p53 or small molecules targeting key signaling interactions involving mutant p53 have been described, including gene therapy that uses viruses to deliver p53 to cancer cells [55]. Among the different strategies, the small-molecule APR-246, able to induce a conformational change toward wild-type like structure [56], has been shown to have strong cytotoxic effects in several cancer cell lines [57–59] and is currently under investigation in patients with various solid tumors [52]. However, these strategies are all in their early clinical development and none of them are currently available.

## 5. Other New Strategies and Neoantigens

Other recent observations point to immunotherapy as a valuable therapeutic approach for ACC. For example, the analysis of nonsynonymous mutations likely represents a useful predictive marker in selecting tumor types that are mostly likely to respond to the immune checkpoint therapy [60, 61]. The mutational load, in fact, is defined as the total number of somatic nonsynonymous point mutations that, by generating novel gene products detected by the immune subsystem as foreign, may trigger an anticancer response [60–63]. On this line, analyses of the mutational load in ACC tumors resulted in an intermediate mutational load value, thus suggesting that ACC could respond to immunotherapy [64].

According to previous conclusions, recent evidences underlined the potential value of microsatellite instability as determinant of immune responsiveness in ACC patients. While in a normal cell, the length of microsatellites is maintained stable during multiple cell divisions by the mismatch repair (MMR) system, in cancer cells, the length of microsatellites can vary due to defects in the MMR system leading to the so-called “microsatellite instability” (MSI). Tumors with abnormal MMR processes and high MSI lead to additive mutations throughout the genome (e.g., “hypermutator” phenotype), a condition that is associated with response to immunotherapy [65]. Bonneville et al. recently found MSI in 4.35% of ACCs, a result which is inferior to that found in classical MSI-high-colon cancer (19.7%), but higher to the median value found across 39 tumor types (3.8%) [65]. Furthermore, high MSI is a constitutional characteristic of the Lynch syndrome, an autosomal dominant genetic condition associated with high risk of colon cancer as well as other cancers including ACC [66]. Recently, mutations in the MUTYH gene encoding for a DNA glycosylase involved in base excision repair (BER) of DNA damage have been described in two series of ACC patients. This finding further expands the mutational asset and MSI of ACC tumors and may, therefore, represent another potential predictive signature of immunotherapy efficacy different from MMR system [67].

The timing of an immune intervention could also play a role in determining its efficacy. Probably, immunotherapy has more chances to be effective in an advanced metastatic ACC rather than in an early one. Recent evidences have in fact highlighted that metastatic ACCs display a higher tumor mutation rate and tumor heterogeneity than primary tumors. Thus, this temporal and spatial heterogeneity could represent a potential advantage for immunotherapy [68].

Finally, the finding of the high expression of the Melan-A/MART1 in ACC [69] which is used as a marker for identifying lesions with adrenocortical origins [18] may also support the notion that ACC would have the chance to respond to immunotherapy against selected neoantigens. This melanoma-associated antigen, in fact, has been described as a human melanoma antigen recognized by autologous cytotoxic T cells [70].

## 6. Conclusions

Results obtained so far hardly lead to considering immunotherapy as a possible immediate therapeutic opportunity for ACC patients. Whether or not immunotherapy will offer a new hope for the management of ACC, however, needs to be further investigated, in particular in a combination therapy, that includes checkpoint inhibitors administered after or in association with chemotherapy molecular target therapies or radiation therapy [71, 72]. Several lines of evidence indicate in fact that the cytotoxic effects of chemo- and radiotherapy may function as immunogenic treatments by inducing expression or reexpression of tumor-associated antigens (TAAs) or by inducing additional new mutations and, therefore, inducing T-cell-specific immune responses [71, 72].

Furthermore, patients that could have the chance to receive clinical benefit from this approach need to be selected also through a molecular approach, in order to obtain a clinical efficacy, with a strict evaluation of the benefit/risk profile. Indeed, we recently proposed to test immunotherapy in ACC patients with altered MMR pathway concomitant with high levels of MSI [73]. Thus, in the future, the potential efficacy of immunotherapy also in the ACC setting will require an accurate patients' selection by means of a genetic approach and a multimodal treatment combining systemic antineoplastic therapies and/or radiotherapy and/or drugs inhibiting steroid synthesis and controlling hypercortisolism.

## Figures and Tables

**Table 1:** Clinical studies investigating immunotherapy in ACC.

Drug	Target	Study phase	Patients	Results	Ref.
Avelumab	PD-L1	I	50	ORR: 6% OS: 10.6 months PFS: 2.6	[16]

Ipilimumab + radiotherapy	CTLA-4	I/II	Active, nonrecruiting	-	NCT02239900

Pembrolizumab	PD-L1	II	Recruiting	-	NCT02673333

Nivolumab + ipilimumab	PD-1/ CTLA4	II	Recruiting	-	NCT03333616

ORR: overall response rate; OS: overall survival; PFS: progression-free survival.
